# Strengthening E-learning strategies for active learning in crisis situations: a mixed-method study in the COVID-19 pandemic

**DOI:** 10.1186/s12909-023-04725-z

**Published:** 2023-10-11

**Authors:** Mohammad-Ali Jahani, Aram Ghanavatizadeh, Sahar Delavari, Mahdi Abbasi, Hossein-Ali Nikbakht, Zeynab Farhadi, Alameh Darzi, Ghahraman Mahmoudi

**Affiliations:** 1 Social Determinants of Health Research Center, Health Research Institute, Babol University of Medical Sciences, Babol, Iran; 2 Hospital Administration Research Center, Sari Branch, Islamic Azad University, Sari, Iran; 3 Institute for the Developing Mind, Children’s Hospital Los Angeles, Keck School of Medicine, University of Southern California, Los Angeles, CA USA; 4 Department of Health Economics and Management, School of Public Health, Tehran University of Medical Sciences, Tehran, Iran; 5 Babol Education Department, Babol, Iran

**Keywords:** E-learning, Active learning, Feedback, COVID-19 pandemic

## Abstract

**Background:**

Medical universities are responsible for educating and training healthcare workers. One of the fields significantly impacted by the pandemic is medical education. The aim of this study is to identify strategies for enhancing e-learning for active learning and finding solutions for improving its quality.

**Methods:**

This mixed-method (quantitative-qualitative) research was conducted in 2023 at three selected universities in Mazandaran Province. In the quantitative phase, 507 students participated via stratified random sampling using a standard questionnaire. In the qualitative phase, data were collected through semi-structured interviews with 16 experts until data saturation was achieved. SPSS 21 and MAXQDA 10 software were used for data analysis.

**Results:**

In the multivariate regression analysis, an increase of one point in the dimensions of student-teacher interaction, active time, immediate feedback, and active learning corresponded to an average increase in learning scores of 0.11, 0.17, 0.16, and 1.42 respectively (*p*≤0.001). After the final analysis in the qualitative phase, four main domains (infrastructure, resources, quantity of education, and quality of education) and 16 sub-domains with 84 items were identified.

**Conclusions:**

The greatest challenge in e-learning is the interaction and cooperation between students and teachers. The implementation of the identified strategies in this research could provide useful evidence for policymakers and educational administrators to implement interventions aimed at addressing deficiencies and enhancing e-learning.

**Supplementary Information:**

The online version contains supplementary material available at 10.1186/s12909-023-04725-z.

## Introduction

### Background

The COVID-19 pandemic, which emerged in 2019, rapidly engulfed many countries. The World Health Organization (WHO) declared it a Public Health Emergency of International Concern (PHEIC) [1]. Despite the cancellation of the PHEIC in May 2023, the pandemic has resulted in approximately 770 million cases and 7 million deaths [2]. Quarantines, long-term restrictions, disruptions in social and economic life, and the exacerbation of social inequality and vulnerability have been only a fraction of its effects [3]. One notable area profoundly affected by the pandemic is healthcare workers’ education. At the outset of the pandemic, many universities worldwide suspended in-person classes and internships [4], forcing students to stay at home to adhere to social distancing protocols [5]. The education system in many countries embraced e-learning methods and accelerated the development of online learning modalities [6].

E-learning has significantly impacted the medical community. For instance, in a survey conducted by Singh et al. approximately 44% of surveyed students believed that online lectures during the pandemic had lower quality than in-person classes [7]. In a study by Shahrvini et al., over 43% of students reported that e-learning did not adequately prepare them for internships [8]. Wanigasooriya et al. found that more than 81% of medical students believed that the pandemic had a negative impact on their education [9]. The reduction in interactions between learners and patients, professors, and fellow students potentially diminishes students' communication skills [10]. Conversely, e-learning has provided benefits such as flexibility in time and location [11], cost-effectiveness [12], positive effects on students' mental health [13], encouraging the use of new technologies [14, 15], and facilitating immediate feedback [16].

In addition, active learning (AL) has always been considered an educational approach to improve students' participation in the learning process and, as a result, improve their knowledge and skills [17]. AL includes a set of interventions that facilitate students' participation in the learning process. These interventions include case-based learning, experiential learning, peer problem solving, project-based learning, etc. [17, 18]. Using AL and moving away from traditional teaching methods can bring better educational results. For example, Freeman et al. showed in their study that the use of AL interventions increases the educational performance of students. They also showed that the probability of students failing in classes with traditional lectures is 1.5 times higher than that of students in classes with AL [18]. Based on this, strengthening e-learning using AL interventions can improve the performance of medical students.

Overall, it appears that the challenges posed by the COVID-19 pandemic have propelled educational systems to adopt e-learning methods as a tool to strengthen medical education [19]. This pandemic has demonstrated the vulnerability of medical education during critical situations [20]. Therefore, establishing a blended learning environment that combines traditional methods with innovative e-learning tools can enhance the responsiveness of the educational system in crisis situations [21]. Additionally, strengthening the infrastructure of e-learning and expanding its application to areas such as student assessment, communication skills, and remote content production should be prioritized [22, 23]. However, e-learning during a pandemic has encountered issues in developing countries, including internet quality deficiencies, security concerns, audiovisual problems, and limited technical skills among students and faculty members [4]. In this regard, curricular reforms that exhibit greater compatibility with e-learning have been recognized as crucial [24]. Given the aforementioned content, conducting applied studies aimed at identifying challenges and strategies for strengthening e-learning in different countries can provide valuable evidence to policymakers, enabling them to employ e-learning as a key strategy for ensuring the continuity of medical education during critical circumstances.

### Objective

The present study was conducted to identify strategies for enhancing e-learning for active learning during crisis situations, such as the COVID-19 pandemic.

## Methods

This study employed a sequential explanatory mixed-methods design, which combines and analyzes both quantitative and qualitative data in a single study, with data collected sequentially. The sequential explanatory method is a two-stage mixed-methods approach in which qualitative data help explain initial quantitative results [25]. The present study consisted of two phases: a quantitative phase examining the quantity and quality of e-learning from the students' perspective, and a qualitative phase exploring the experiences of experts during the COVID-19 pandemic and strategies for enhancing e-learning for active learning in crisis situations.

### Quantitative phase, examination of the quantity and quality of e-learning from students' perspective

#### Participants

The target population for the quantitative phase comprised 13,500 students from medical universities in Mazandaran province. The recommended sample size ratio ranges from 5 to 15 variables per sample (5q ≤n≤15q) where q represents the number of observed variables or questionnaire items, and n represents the sample size [26]. In the present study, the sample size for the quantitative phase was determined to be between 480 and 520 individuals. A cluster sampling method was used. The inclusion criterion was students from selected universities who had participated in e-learning courses.

#### Measurements

The data collection instrument in the quantitative phase was a questionnaire that was initially developed in Turkey by Onal Cakiroglu in 2014. The English version of the questionnaire was validated by Onal Cakiroglu and colleagues [27]. The questionnaire consisted of 40 items assessing the quality of e-learning in the dimensions of interaction, teaching, and learning across seven domains. Interaction is related to the relationship between faculty and student and includes two dimensions encouraging student-faculty contact and encouraging cooperation among students. Learning, refers to the learning process in students and the role of faculty members in it. This dimension has two domains encouraging active learning and respecting diverse talents and ways of learning. Finally, teaching is related to the process teaching of faculty members and has three dimensions: giving prompt feedback, emphasizing time on tasks and communicating high expectations.

The questions were rated on a five-point Likert scale, ranging from “Very Low” (1) to “Very High” (5). Therefore, score between 1 and 1.80 indicated very poor quality, between 1.81 and 2.60 indicated poor quality, between 2.61 and 3.40 indicated average quality, between 3.41 and 4.20 indicated good quality, and above 4.21 indicated very good quality of e-learning. The Persian version of the questionnaire was validated and its reliability was assessed in Iran by Ganavati Zadeh et al. [28].

#### Data analysis

Data analysis was performed using IBM® SPSS® Statistics 21. Descriptive statistics such as frequencies, percentages, means, and standard deviations were calculated. Furthermore, both univariate and multivariate linear regression analyses were conducted at a significance level of *p* ≤ 0.05 to examine the relationships between variables.

### Qualitative phase: strategies for enhancing E-learning in crisis conditions

#### Participants

The research population in the qualitative phase consisted of research experts in medical education. The inclusion criteria were their involvement in planning, management, and executive activities related to e-learning during the COVID-19 pandemic, as well as their prior experience in e-learning. Purposive sampling was used in the study. In total, 16 experts who met the criteria of the study target group participated in the study.

#### Measurements

Semi-structured interviews were conducted to gather data in this phase. The interview guide and questions were developed based on the analysis of the quantitative phase results, as well as the experiences of experts during the pandemic. Consequently, after analyzing the quantitative phase, the questions that received the lowest scores from the students' perspective in the questionnaire were identified. The interview questions focused on the main challenges of medical education and barriers to active learning during the pandemic, as well as strategies for enhancing active learning in crisis situations.

#### Analysis

Data in the qualitative phase were analyzed using the content analysis method with the assistance of MAXQDA 10 software. Braun and Clarke's thematic analysis approach was employed, involving familiarization with the data, initial coding, searching for themes, reviewing themes, defining themes, and preparing a report for data analysis [29]. The interview content was transcribed, and multiple reviews were conducted to extract initial codes for a comprehensive understanding. The initial codes related to strategies for enhancing e-learning were identified within the interview texts. Similar codes were grouped into sub-themes and further organized into main themes. To enhance the validity of the data, researchers employed strategies such as allocating sufficient time for interviews, examining the topic from various perspectives, obtaining research validation from experts, involving two coders for interview coding, seeking peer debriefing, and sharing findings with some of the interviewees.

## Results

### Quantitative phase results

The average age of the participating students in the study was 21.47 ± 2.34, with an age range of 18 to 43 years. The majority of participants were female (62%, *n* = 319) (Table 1).

**Table 1:** Examination of the demographic characteristics of the participating students

Variable	Groups	Frequency	Percentage
Gender	Male	188	37.08
	Female	319	62.92
Age Group	Less than 23 years	421	83.04
	23 and above	86	16.96
Educational Level	Bachelor's degree	279	55.03
	Master's and Professional Doctorate	204	40.24
	Residency	24	4.73
Academic Year	First-year	113	22.29
	Second year	161	31.75
	Third year	193	38.07
	Fourth-year and above	40	7.89

The descriptive statistics of the scores for the axes and dimensions of the questionnaire on a standard scale of 0 to 100, for comparison of axes and dimensions in the same unit, indicate that the highest scores by students were for the Immediate Feedback and High Expectations axes, with scores of 64.65 and 65.46, respectively. The lowest score was for the Collaboration Among Students axis with a score of 55.90 (Table 2).

**Table 2:** Average scores of the studied universities in relation to online teaching based on the extent of axes and dimensions of active learning during the COVID-19 pandemic (standard scale 0–100)

Axis and Dimensions		Number of Questions	Score Range	Mean (S D)	Mean Score (0–100)
Axis	Student Interaction with Instructor	6	6–30	17.77(5.28)	59.23
	Collaboration among Students	6	6–30	16.77(3.79)	55.90
	Timing of Activities	6	6–30	17.87(4.18)	59.56
	Immediate Feedback	5	5–25	16.41(3.30)	65.64
	Active Learning	6	6–30	18.42(4.47)	61.40
	High Expectations	6	6–30	19.64(4.40)	65.46
	Diverse Abilities and Learning Methods	5	5–25	15.38(4.12)	61.52
Dimension	Interaction	12	12–60	34.54(8.23)	57.56
	Teaching	17	17–85	53.93(10.15)	63.44
	Learning	11	11–55	33.80(8.01)	61.45

The results of the linear regression test in separately examining the relationship between the Teaching dimension and the Interaction and Learning dimension axes showed that for each increase in score in the High Expectations axis, Diverse Talents axis, and Learning Methods axis, the Teaching dimension score increases significantly by 2.00 and 1.91, respectively (*P*≤0.001). In the multivariate regression analysis related to these factors, the results showed that all axes of the Interaction and Learning dimensions are associated with the Teaching dimension score. Therefore, on average, for every unit increase in the score on the High Expectations, Diverse Talents, and Learning Methods axes, an increase of 1.30 and 0.44 units in the Teaching dimension score is observed respectively (*P*≤0.001) (Table 3).

**Table 3:** Relationship between the teaching dimension and the sub-dimensions of the interaction and Learning dimensions

variables	Univariate Analysis (Raw Effects)			Multivariate Analysis (Adjusted Effects)		
	B(SE)	%95 CI	*P*-value	B(SE)	%95 CI	*P*-value
**Student interaction with instructor**	1.38 (0.05)	1.26 to 1.49	0.000	0.39 (0.05)	0.28 to 0.49	0.000
**Collaboration among Students**	1.60 (0.09)	1.41 to 1.79	0.000	0.23 (0.06)	0.01 to 0.36	0.000
**High Expectations**	2.00 (0.05)	1.90 to 2.10	0.000	1.30 (0.06)	1.17 to 1.43	0.000
**Diverse Abilities and Learning Methods**	1.91 (0.06)	1.77 to 2.04	0.000	0.44 (0.07)	0.03 to 0.58	0.000

Additionally, the regression analysis was conducted to examine the relationship between Interaction with the subdimensions of Teaching and Learning, and the relationship between learning and the subdimensions of interaction and teaching. In one case, the collaboration among students was not significant (*P*=0.262), and in other cases, there was a significant correlation (*P*<0.001) (Supplementary Table 1).

### Qualitative phase results

All participants were members of the university faculty, with 9 individuals (56.25%) being male, 4 (25%) being associate professors, and the rest being assistant professors. Six (37.5%) were clinical faculty members and the rest were in basic sciences. All had at least 5 years of teaching experience and had planning and executive responsibilities during the COVID-19 pandemic.

Following the final analysis of online teaching by experts, four main domains were identified, including infrastructure, resources, quantity of education, and quality of education, and 16 subdomains, namely, scientific infrastructure, technical infrastructure, communication infrastructure, technology infrastructure, resource savings, increase in personal expenses, increase in university expenses, educational content, timing, continuous education, student participation, interaction, feedback, practical classes, and evaluation, with a total of 84 items generated (Table 4).

**Table 4:** Main domains, sub-domains, items, and their frequency in online teaching as perceived by experts

Main domain	Subdomains	Issues	Occurrence of Issues
**Infrastructure**	**Scientific Infrastructure**	Uploading educational content	16
		Increasing capabilities of teachers after experiencing virtual teaching and working with various educational software	16
		The unpreparedness of teachers for online and virtual teaching	15
		The unpreparedness of students for online and virtual learning	15
		Learning to work with the system through trial and error by teachers	12
		Continuous collaboration of university technology colleagues with teachers	12
		Conducting educational sessions to enhance the capabilities of teachers	10
		Resistance to virtual teaching and learning by some teachers and students at the beginning of the COVID-19 pandemic	7
	**Technical Infrastructure**	Availability of limited technical infrastructure in universities before the pandemic	16
		Challenges in improving the technical infrastructure of universities and educational medical centers after the start of virtual teaching	13
		Lack of suitable hardware for this method of teaching and learning by teachers and students	11
		Existence of executive regulations for online teaching implementation prior to the pandemic to some extent	10
		Lack of compulsion by the university regarding virtual teaching by teachers before the pandemic	10
		Incompatibility of the existing platform with the number of students using it during the pandemic	10
		Establishment and equipping of a virtual examination center at the university	7
		Establishing adequately equipped rooms for producing standardized virtual content at the university	7
		Lack of coordination between existing regulations and changes after the pandemic	6
	**Communications Infrastructure (Internet)**	Lack of suitable internet with the desired bandwidth	16
		Power outages followed by internet disconnections	16
		Lack of access to suitable internet in various parts of the country	12
		Increasing the internet bandwidth of the university for coordination with the current conditions	7
	**Technological Infrastructure**	Ability to upload assignments in audio and video formats	16
		Specifying assignment titles	16
		Ability to modify and edit uploaded assignments	16
		Ability to access uploaded files	16
		Conducting online exams, both in multiple-choice and descriptive formats	16
		Monitoring teaching methods and professors' attendance and departure time	12
		University supervision over files uploaded by professors	10
**Resources**	**Resource Savings**	Reduced costs for teachers and students due to less commuting and mobility	13
		No educational limitations due to the geographical distance of students from the university	13
		Participation in scientific seminars of various domestic and international universities without travel expenses, in the form of webinars	11
		Cost reduction and reduction in air pollution through less commuting and mobility	6
	**Increased Personal Expenses**	Increased personal expenses due to the purchase of necessary equipment and facilities for virtual teaching for both teachers and students	11
		Increased personal expenses due to the purchase of internet packages	10
	**Increased University Expenses**	Procurement of necessary equipment and supplies for online teaching	8
		Increased expenses to improve the online teaching environment	7
		Construction and equipping of a virtual examination center at the university	7
		Construction and adequate equipment of rooms for producing standardized virtual content at the university	7
		Increasing the internet bandwidth of the university for coordination with the current conditions	7
**Quantity of Education**	**Educational Content**	Preparation and uploading of simple educational content at the beginning of COVID-19 and offline education to coordinate with the current conditions	7
		Necessity of preparing audio and video educational materials by teachers	7
		Ability to create a package of prepared video tutorials in the faculty library	7
	**Timing**	Possibility to increase teaching time in theoretical subjects	13
		Scheduling classes based on teachers' duties and responsibilities	12
		Increased teaching time through the supervision of the education unit on the teachers' teaching via the system	12
		Increased teaching time due to less student participation	10
		Limited timing for online class formation due to the capabilities of the existing platform	9
		Inability to add class time even for a few minutes after the specified time in the system	6
	**Ongoing Education**	Continuous access of students to educational content	16
		Possibility of online teaching during class holidays	14
		Possibility of holding online classes outside of working hours	12
**Educational Quality**	**Student Participation**	Decreased student participation	14
		Dissatisfaction of teachers with student participation	14
		Lack of student control	14
		Possibility of passive attendance	12
		Difference in student participation in different academic levels	10
		Precautionary measures by some teachers	6
	**Interaction**	Less interaction in online teaching	16
		Inability to have multi-person conversations on the platform due to its limitations	16
		One-way communication on the existing platform	12
		Written or verbal conversations	11
		Excuses from students regarding non-participation in conversations	11
		Differences in the approach of different teachers in creating an open environment in online classes	10
		Expressing opinions and views by students for various reasons	10
		Possibility of freely expressing opinions by students throughout the online class	9
		Need for teachers to be aware of the impact of student opinions on the learning process	8
		Less interaction in students who had virtual education from the beginning of entering the university compared to senior students	7
		Ability to create interactive content to increase interaction in online teaching	5
	**Feedback**	Various methods of receiving and providing feedback from teachers to students	15
		Feedback provided to students by teachers	14
	**Access**	Easy access through social networks	12
		Teacher conditions	8
		Reduced access in online teaching	6
	**Practical Classes**	Maintaining patient confidentiality	8
		Insufficient skill acquisition in practical and clinical subjects online	8
		Decreased learning in practical and clinical subjects for students	8
		Inability to procure necessary equipment for online teaching in practical and clinical subjects due to high costs	7
		Reduced duration of clinical and practical classes	7
	**Evaluation**	Lack of trust of teachers in the teaching method	16
		Significant difference in students' grades between online and in-person teaching	16
		Unpreparedness of teachers and students for evaluation	15
		Challenges in entering questions by teachers in the system	13
		Use of process-oriented evaluation method instead of result-oriented evaluation	13
		Reduction in the question bank of some courses due to students saving questions	7

#### Infrastructure

##### Scientific infrastructure

Participants in the study pointed to the importance of online teaching and the need for preparedness for e-learning in teachers and students and their capabilities. Participant 3 emphasized the importance of having a scientific infrastructure for online teaching and the necessity for teachers' preparedness, saying, “*The university did not provide any training for us, and I learned to work with the system through trial and error. It took me a few sessions to learn how to work with e-learning*”. Additionally, participant 8 talked about the lack of complete readiness of teachers for online teaching before the COVID-19 pandemic, despite the presence of guidelines for using this method in teaching, “*The university had been talking about e-learning for four or five years ago, set up classes, but because it was not mandatory, we did not attend. Personally, I did not go, and I hadn't worked with its software. Well, the year that COVID-19 emerged and classes became virtual, the first thing I did was read the guide that existed and I learned by trial and error*”.

##### Technical infrastructure

Technical infrastructure problems in universities and educational and therapeutic centers, and the incompatibility of the existing platform with the volume of students, were the issues that the experts pointed to. Participant 1 said, “*The infrastructure was present in our college, considering we had a virtual unit before, but it wasn't enough. The main challenge was the lack of a tablet or laptop or a mobile device to run classes, which was both for teachers and students*”.

##### Communication infrastructure

Issues related to internet disconnection and inadequate access in various geographical areas were noted in this area. Participant 16 emphasized the undeniable impact of internet quality on all aspects of online education and teaching, adding, “*One of the main challenges was internet disconnection. For instance, it would suddenly get cut off in the middle of the class and by the time it got reconnected, the class time would be over. The next professor would arrive with the subsequent class, and then we were forced to hastily cover the leftover topics from the previous class at the start of the next session*”.

##### Technology infrastructure

Software capabilities and actions that learners can undertake through this type of learning were among the topics to which experts referred. Participant 5 spoke about the execution of assigned tasks by students and their uploading onto the electronic system, stating, “*A good thing that was done was the tasks we gave to the students, like writing an article with the rules we set for them, or researching a specific topic. After the student uploaded it, we could easily receive them and then evaluation could be done*”.

#### Resources

##### Saving resources

Experts referred to lower teaching costs, commuting, and the possibility of participating in scientific webinars virtually, etc. On this matter, Participant 6 stated, “*In my opinion, one of the advantages and opportunities of online teaching is the reduction of costs because neither we nor the students needed to move from our workplace or residence to hold or participate in classes*”. Participant 14 mentioned this method as an opportunity to participate in scientific seminars that are held as webinars at various universities in the country, saying, “*During this period, we could easily participate in and benefit from various educational webinars held by different universities across the country*”.

##### Increase in personal expenses

The shift from face-to-face to e-learning necessitated the provision of certain facilities, leading to an increase in the personal expenses of learners and teachers. Regarding the necessity of certain facilities for both teachers and students to participate in and hold virtual classes, Participant 5 said, “*Naturally, to hold and participate in online classes, some equipment such as a laptop, tablet, or smartphone is needed, which some teachers and students did not have at the beginning and had to acquire. On the other hand, we always needed to be connected to the internet, all of which led to an increase in personal expenses*”.

##### Increase in university expenses

The shift from face-to-face to e-learning and the need to provide facilities also burdened universities with increased costs. Participant 10 added regarding the need for some expenses to be borne by universities to improve the online teaching environment during the COVID-19 pandemic: “*Initially, the university's facilities were very simple and limited, and we had to either procure some facilities from scratch or upgrade our existing ones to address some deficiencies and improve the online teaching environment, and subsequently, improve the quality of learning*”.

#### Quantity of education

##### Educational content

The ability for teachers to create audio and visual educational content and make it accessible was one of the points mentioned. Participant 7 said, “*Initially, materials were uploaded as theory and PDF, but some lessons were such that students could not grasp the content with PDF alone. Various software was used, for example, anatomy teachers would come to the anatomy room, film, cut the videos into pieces, and then use software to reduce the size*”.

##### Scheduling

Attention to time, and the advantages and limitations of e-learning in terms of scheduling, were among the points mentioned: Participant 15 talked about the time limit of the classes in addition to the scheduling limit: “*One of the challenges is the limitation and timing of the classes. For instance, a class that I wanted to last more than two hours was not feasible and the connection and session would be cut off without my intention. I had to hold the class at another time when I was busy*”.

##### Continuous learning

E-learning made it possible to teach outside of obligatory times. Participant 8 discussed the opportunity for online teaching at times when there is a possibility of class cancellations, for the continuation of teaching and continuous learning, “*In my opinion, it is a good opportunity. For instance, when there are a few days off or one or two days between holidays, usually students from other cities cancel the class to take the opportunity to go home*”.

#### Quality of education

##### Student participation

Overall, it was stated that student participation in e-learning was less than in-person teaching. (Participant 9) said that student participation in online teaching was less than that in traditional in-person teaching, “*The participation in this teaching was less, and I asked questions to keep the students in class. For example, I would ask a short answer question and ask them to answer, or during attendance time or students' breaks, I would tell the students not to leave the classroom, and use attendance as an excuse*”.

##### Interaction

In this regard, the experts gave their views on interaction in e-learning: Participant 11 continued with the idea that interaction in online teaching is less than traditional in-person teaching: “*If I want to say this honestly, it is better in-person, because in online teaching, you can only be sure of students' online presence, but real interaction and presence is not recorded*”. Participant 10 suggested creating interactive content to increase interaction in online teaching, “*We can't have good interaction in online classes, perhaps by creating interactive content such as storylines*”. Participant 12 discussed the difference in interaction in online teaching compared to in-person teaching: “*It was less, obviously less. Because the time was limited, the space was limited and there was no visibility. For instance, interest, enthusiasm, that face-to-face connection was not seen, it makes a big difference*”.

##### Feedback

Regarding the possibility of feedback in e-learning, it was mentioned that feedback to students from teachers was available, but it might not always be immediate and online for various reasons. Participant 9: “*Feedback was there during the class or in the form of sending a message to my personal number to the students, but sometimes I might not be able to respond online*”. Participant 1 spoke about various methods of receiving and sending teacher feedback to students: “*We could give feedback to students in various ways, such as through educational platforms during online classes or through other available messaging systems*”.

##### Access

Regarding the different ways students can access teachers, Participant 14 said: “*Now there are many ways to access teachers because they are usually present in all virtual system tools, they have an ID, they have a page and all that, and I do not think there is much difference between in-person and online teaching in this regard*”.

##### Practical classes

The experts pointed to the weakness of learning practical classes through online education. Participant 14 stated, “*Generally, clinical work is a very big challenge in online subjects, and we cannot teach all clinical cases online. We cannot do this because one of the reasons is the issue of patient confidentiality*”. Participant 10 also said, “*Some practical lessons, for example in the laboratory, cannot be taught virtually and online. Let us assume that we put a video for them to see, can we say that they learned the material?*”

##### Evaluation

Participant 9 talked about the problems related to holding exams and the lack of readiness of teachers and students in this regard: “*Usually, we assigned part of the grade to the tasks we gave to students, which again were not very reliable because someone else could have done it for them, but we had to trust them*”. Participant 8 spoke about the problems related to online exams: “*First, they had informed us at the time of the online exam that the students were gathering in groups and were taking the exam together and consultatively, and on the other hand, they were taking screenshots of the questions. Given the nature of the educational content, there is a limitation of questions in some topics, and this way, our entire question bank was leaked*”.

### Strategies for enhancing active learning

One of the solutions that participants referred to was the pathology of e-learning. An analysis of approximately three years of university performance in using e-learning and identifying weaknesses can be the most important solution for strengthening e-learning. Participant 8 “*Given that universities and stakeholders were not prepared for this pandemic, the sudden and emergency use of e-learning naturally faced numerous challenges and weaknesses. However, the crucial point is that the medical education system should have the necessary resilience for the next crisis situation. To achieve this goal, identifying weaknesses and damages that our e-learning has had during this period is very essential*”. Participant 11: “*The important point is that the education system's readiness to deal with crisis situations should be constantly monitored and evaluated and not be limited to a short period after the pandemic*”.

Another solution identified in this research is improving internet quality. Most interviewees believed that poor internet speed has been a fundamental problem in e-learning. Participant 16: “*Some students living in remote areas and having less access to appropriate internet had doubled e-learning problems*”, Participant 1: “*This issue even affected educational equity as these students could not effectively participate in classes*”.

Producing effective educational content is another solution to strengthen e-learning. Accordingly, it is necessary for faculty members to use diverse and attractive content to achieve the goal of enhancing students' learning. (Participant 7): “*Over time, professors moved toward producing varied content, including films, slides, podcasts, sounds, clips, etc. For instance, anatomy professors would go to the dissection room and film, then break the films into pieces and use special software to reduce their size, and upload them to the system*”.

The use of blended learning methods combining in-person and virtual instruction was proposed to enhance student evaluation. One of the participants stated, “*The primary issue with e-learning was assessing the students while minimizing the chance for student dishonesty and cheating. To address this problem, I usually allocated a portion of the score to students' assignments”.*

The use of process-oriented evaluation was another solution proposed by participants in this research. “*Continuous and ongoing evaluation can reduce the challenges of virtual assessment. Part of the students' final score could be based on assignments and activities throughout the term, and the rest based on the end-of-term exam*”.

Based on participants' feedback, the lack of guidelines and instructions for e-learning was one of the main challenges when e-learning became mandatory. Consequently, expediting the process of preparing and publishing e-learning guidelines, as well as their constant review, were identified as some of the most significant proposed solutions. Another proposed solution is to continue blended learning even after the pandemic ends. Participants believed that the quality of teaching could be improved through e-learning if it is used alongside in-person instruction. However, opinions varied regarding its continuation. Some professors suggested that a specific percentage of each course's sessions be held virtually. Another perspective was to use e-learning to provide supplementary educational content and assign students tasks. Some participants proposed that theoretical educational materials be prepared as standard educational content and provided to students before class. Then, during in-person classes, this content should be analyzed and discussed for deeper learning.

One of the main challenges of e-learning is the interaction between the teacher and the student. Many interviewees suggested that interventions aimed at enhancing teacher-student interaction are crucial for improving the quality of e-learning. To increase interaction with students, some professors used strategies such as question-and-answer sessions, inviting student participation, assigning grades for student engagement, and creating interactive content. (P8): “*To encourage participation, I would call out a student's name and ask for their opinion. However, some students claimed that their microphone was not working, or the sound was not coming through, or they would give similar excuses*”. In addition, identifying and utilizing e-learning systems that offer numerous measures to engage students can be helpful.

Many interviewees believe that adequate acculturation regarding the proper use of online education for all stakeholders, including students and their families, teachers, education managers, and experts, is crucial. Some participants considered the lack of proper acculturation as the main reason for resistance from some teachers and students against the use of the e-learning platform. Participant 7 stated, “*In the beginning of using online education, some students were unable to participate effectively in the classes due to lack of cooperation from their families*”. Finally, the success of each of the above solutions requires the management of resources and infrastructure. Almost all interviewees identified the lack of equipment and infrastructure as problematic. For example, one of the professors (Participant 14) stated, “*Initially, we had problems both in electronic communication infrastructure and equipment. However, fortunately, this challenge turned into an opportunity. Because universities had to invest to rectify the deficiencies. Afterwards, with the utilization of this, all treatment centers were equipped with e-learning systems and educational equipment*”.

## Discussion

The quantitative findings showed that the most significant issues with online education were student cooperation (2.79) and student-teacher interaction (2.96). These results align with the study by Çakýroðlu and colleagues. Their research revealed that, from the perspective of selected students in Turkey, the weakest performance of online education was in the axis of student cooperation (2.59 out of 5 points) [27]. In a study by Kim et al., 62% of students were dissatisfied with the lack of interaction between the student and the teacher in online education. Interestingly, only approximately one-third of teachers believed there was inadequate interaction with students [30]. These results indicate that teachers do not have a full understanding of students' expectations regarding student-teacher interaction. Furthermore, students may have failed to communicate their expectations to their teachers. Clearly, stating the expectations of students and teachers, organizing brainstorming sessions, and encouraging active student participation can bridge the gap in perspectives. Student-teacher interaction is one of the main limitations created in online education. Abbasi and colleagues demonstrated in their study that 84% of medical science students believed that online education limits the interaction between students and teachers [31]. Parker suggests that one reason for inadequate interaction in online education is the lack of understanding and professional development among some teachers. He believes that the teacher should take on the role of a “stimulator” rather than an “academic.” Accordingly, course design should be altered to incentivize student interaction. Using tools such as humor and sharing real-life stories can create a sense of intimacy that enhances interaction [32].

Other findings from this research showed that activity time and immediate feedback are independent and robust predictors for the interaction dimension. “Activity time,” means that teachers give students enough time to perform activities and assignments so they can complete them at a leisurely pace without running out of time [27]. In the present study, over 57% of participants were dissatisfied with the course schedule and timing set by teachers. Proper planning by teachers for activity completion is crucial. Teachers should, with a suitable plan, set appropriate deadlines for students to complete their activities, taking into account potential problems and giving students enough time. Timely feedback from students and teachers and analysis of feedback improves the effectiveness of online learning and teaching [33]. In the present study, among the axes of e-learning, students gave the highest score to immediate feedback. However, more than half of the participants (53.8%) believed that feedback from classmates and teachers was delayed. Teachers can use various methods such as online education systems, email, and social networks to provide feedback. Additionally, teachers should ensure that students receive their feedback.

In the quality phase, solutions for strengthening e-learning included e-learning pathology, improving internet quality, preparing diverse educational content, using physical and virtual student assessment, developing practical guidelines, equipment management, culturalization, continued use of blended education after critical conditions, using process-based assessment methods, and strengthening student and teacher interaction. Similar solutions have been identified in other studies. For example, Dost et al., considering the dissatisfaction of approximately half of the students with the quality of the internet, suggested improving its quality as a fundamental solution [34]. Nimavat et al. also suggested solutions such as culturalization, empowering teachers, effective time management, and managing equipment and infrastructure [33].

Providing the necessary infrastructure for e-learning is a critical step in strengthening it. In many developing countries, a lack of resources and infrastructure has been a major problem in the path to virtualizing medical education [5, 28, 35]. However, the outbreak of the COVID-19 pandemic has provided an opportunity for governments to create the necessary infrastructure through investment [33]. However, this problem persists in some countries [36] and solving the problem requires attention to this issue in countries' economic policies, and financial and equipment assistance from international institutions and advanced countries to underdeveloped countries.

The development of efficient guidelines can also accompany the strengthening of e-learning. The insufficient technical skills of teachers and students in using the e-learning platform have been a fundamental challenge during the COVID-19 pandemic. This negatively affects online education delivery [33]. Technical guides and instructions can be a basis for training teachers and students. Some of the interviewees in the present study believed that improving the quality of e-learning requires the participation of various education sectors, including information technology, education, registration, exams, resource management, and so on, and coordinating between these sectors requires developing transparent and clear instructions. However, it is essential that the guidelines be updated regularly with changing conditions. Access to these guidelines should also be facilitated.

Cultural development is another solution proposed in this study. The resistance of students and professors is a significant challenge for e-learning in countries. Cultivating an understanding of the necessity and applications of e-learning can reduce this resistance. Furthermore, ethical and uncivil abuses have caused some students and professors to feel less secure during e-learning [31]. On the other hand, reports indicate student restrictions by families to participate in virtual classes [33]. To address these challenges, cultural development could be a useful solution.

The use of a combined evaluation of physical and virtual presence is another proposed solution in this study. Using purely virtual evaluations raises concerns for professors about potential abuses by students during examinations [37]. To alleviate these concerns, many universities use a hybrid approach for student evaluation. For example, in Indonesia, online examinations focusing on the camera's transparency were used to evaluate students' cognitive performance. For practical skills assessment, they used a hybrid OSCE exam. Students were present on site at examination stations, while examiners observed the performances live through cameras and online conferences [38]. Utilizing other innovations, such as conducting a portion of the course's exam in person and part virtually, using simulators, and switching from final evaluations to formative assessments can strengthen this dimension of e-learning [33].

## Limitations of the study

The population of this study was limited to medical students of medical sciences universities in Mazandaran province. On the other hand, we evaluated all educational levels together and considering that the challenges are different in different educational levels and in different fields and in different universities, it may affect the overall results.

## Conclusions

In conclusion, the solutions identified in this study, if accompanied by structural and process modifications in e-learning, can have the necessary effectiveness and sustainable changes. Stakeholder commitment is a key prerequisite for the success of these solutions. Therefore, the participation of all stakeholders, including administrators, professors, students, and families, should be sought in their implementation.

In this vein, conducting similar studies in different fields can create a more accurate understanding of the challenges of e-learning in the country and solutions for strengthening it. It is also recommended to analyze and study the challenges of e-learning from the perspective of other stakeholders such as educational administrators and student families.

### Supplementary Information

**Additional file 1: Appendix Table 1.** Relationship between the interaction dimension with the sub-dimensions of the teaching dimension and the learning dimension. **Appendix Table 2.** Relationship between the learning dimension with the axes of the interaction dimension and the teaching dimension.

## Data Availability

The datasets used and/or analyzed during the current study are available from the corresponding author upon reasonable request.
