# Supplementary material for: Strengthening E-learning strategies for active learning in crisis situations: a mixed-method study in the COVID-19 pandemic
Source: BMC Med Educ. 2023 Oct 11;23:754. doi: 10.1186/s12909-023-04725-z (PMC10568816; doi:10.1186/s12909-023-04725-z)
Supplement: Supplementary file 1 — Additional file 1: Appendix Table 1. Relationship between the interaction dimension with the sub-dimensions of the teaching dimension and the learning dimension. Appendix Table 2. Relationship between the learning dimension with the axes of the interaction dimension and the teaching dimension. [file 12909_2023_4725_MOESM1_ESM.docx]

**Appendix Table 1: Relationship between the interaction dimension with the sub-dimensions of the teaching dimension and the learning dimension**

| variables | Univariate Analysis (Raw Effects) | | | Multivariate Analysis (Adjusted Effects) | | |
| --- | --- | --- | --- | --- | --- | --- |
|  | B(SE)* | %95 CI** | P-value*** | B(SE)* | %95 CI** | P-value*** |
| Time of activities | 1.25(0.06) | 1.38 to 1.12 | 0.000 | (0.07) 0.58 | 0.73 to 0.43 | 0.000 |
| Immediate feedback | 1.55(0.08) | 1.72 to 1.38 | 0.000 | (0.10) 0.45 | 0.66 to 0.24 | 0.000 |
| Active learning | 1.09(0.06) | 1.25 to 0.96 | 0.000 | (0.08) 0.22 | 0.38 to 0.05 | 0.008 |
| High Expectations | 1.17(0.06) | 1.29 to 1.04 | 0.000 | (0.08) 0.28 | 0.46 to 0.11 | 0.001 |
| Diverse Abilities and Learning Methods | 1.29(0.06) | 1.42 to 1.16 | 0.000 | (0.10) 0.30 | 0.50 to 0.09 | 0.005 |

**Appendix Table 2: Relationship between the learning dimension with the axes of the interaction dimension and the teaching dimension**

| variables | Univariate Analysis (Raw Effects) | | | Multivariate Analysis (Adjusted Effects) | | |
| --- | --- | --- | --- | --- | --- | --- |
|  | B(SE)* | %95 CI** | P-value*** | B(SE)* | %95 CI** | P-value*** |
| Student interaction with instructor | (0.05) 0.95 | 1.06 to 0.85 | 0.000 | (0.03) 0.11 | 0.18 to 0.05 | 0.000 |
| Collaboration among Students | (0.07) 1.19 | 1.34 to 1.03 | 0.000 | (0.03) 0.04 | 0.12 to 0.03- | 0.262 |
| Time of activities | (0.06) 1.13 | 1.27 to 1.00 | 0.000 | (0.03) 0.17 | 0.24 to 0.10 | 0.000 |
| Immediate feedback | (0.08) 1.59 | 1.75 to 1.43 | 0.000 | (0.04) 0.16 | 0.26 to 0.07 | 0.000 |
| Active learning | (0.02) 1.68 | 1.73 to 1.62 | 0.000 | (0.03) 1.42 | 1.49 to 1.36 | 0.000 |
